# Xanthine Oxidase-Induced Inflammatory Responses in Respiratory Epithelial Cells: A Review in Immunopathology of COVID-19

**DOI:** 10.1155/2021/1653392

**Published:** 2021-08-05

**Authors:** Irandi Putra Pratomo, Dimas R. Noor, Kusmardi Kusmardi, Andriansjah Rukmana, Rafika I. Paramita, Linda Erlina, Fadilah Fadilah, Anggi Gayatri, Magna Fitriani, Tommy T. H. Purnomo, Anna Ariane, Rudi Heryanto, Aryo Tedjo

**Affiliations:** ^1^Department of Pulmonology and Respiratory Medicine, Faculty of Medicine, Universitas Indonesia, Jakarta, Indonesia; ^2^COVID-19 Task Force–Pulmonology and Respiratory Medicine Unit, Universitas Indonesia University Hospital, Universitas Indonesia, Depok, Indonesia; ^3^Bioinformatics Core Facilities, Indonesian Medical Education and Research Institute, Faculty of Medicine, Universitas Indonesia, Jakarta, Indonesia; ^4^Human Cancer Research Center, Indonesian Medical Education and Research Institute, Faculty of Medicine, Universitas Indonesia, Jakarta, Indonesia; ^5^Department of Pathology Anatomy, Faculty of Medicine, Universitas Indonesia, Jakarta, Indonesia; ^6^Drug Development Research Cluster, Indonesian Medical Education and Research Institute, Faculty of Medicine, Universitas Indonesia, Jakarta, Indonesia; ^7^Department of Microbiology, Faculty of Medicine, Universitas Indonesia, Jakarta, Indonesia; ^8^Department of Medical Chemistry, Faculty of Medicine, Universitas Indonesia, Jakarta, Indonesia; ^9^Master's Programme in Biomedical Sciences, Faculty of Medicine, Universitas Indonesia, DKI Jakarta, Depok, Indonesia; ^10^Department of Pharmacology, Faculty of Medicine, Universitas Indonesia, Jakarta, Indonesia; ^11^Universitas Indonesia University Hospital, Universitas Indonesia, Depok, Indonesia; ^12^Division of Rheumatology, Department of Internal Medicine, Faculty of Medicine, Universitas Indonesia–Dr. Cipto Mangunkusumo Hospital, Jakarta, Indonesia; ^13^Department of Chemistry, Faculty of Mathematics and Natural Sciences, IPB University, Bogor, Indonesia; ^14^Tropical Biopharmaca Research Center, IPB University, Bogor, Indonesia

## Abstract

Xanthine oxidase (XO) is an enzyme that catalyzes the production of uric acid and superoxide radicals from purine bases: hypoxanthine and xanthine and is also expressed in respiratory epithelial cells. Uric acid, which is also considered a danger associated molecule pattern (DAMP), could trigger a series of inflammatory responses by activating the inflammasome complex path and NF-*κ*B within the endothelial cells and by inducing proinflammatory cytokine release. Concurrently, XO also converts the superoxide radicals into hydroxyl radicals that further induce inflammatory responses. These conditions will ultimately sum up a hyperinflammation condition commonly dubbed as cytokine storm syndrome (CSS). The expression of proinflammatory cytokines and neutrophil chemokines may be reduced by XO inhibitor, as observed in human respiratory syncytial virus (HRSV)-infected A549 cells. Our review emphasizes that XO may have an essential role as an anti-inflammation therapy for respiratory viral infection, including coronavirus disease 2019 (COVID-19).

## 1. Introduction

Uric acid (UA) is a Danger Associated Molecule Pattern (DAMP) released in stress conditions and is associated with nonspecific inflammation response [1] or neutrophils recruitment to the inflammation sites [2]. Uric acid can activate NALP3 inflammasomes, causing neutrophilic inflammation and interleukin (IL)-1*β* proinflammatory cytokine release [3, 4]. In primates, uric acid is catalyzed by xanthine dehydrogenase (XDH) and xanthine oxidase (XO) [5]. Xanthine dehydrogenase, which is expressed in various tissues such as the liver and intestine, is also associated with the increase of proinflammatory cytokines, such as IL-1*β*, tumor necrosis factor (TNF)-*α,* and interferon (IFN)-*γ* [6, 7].

Xanthine oxidase oxidizes xanthine with the aid of molybdenum cofactor by transferring the electron via two Fe–S clusters to the flavin adenine dinucleotide (FAD) coenzyme part to reduce oxygen and reduce nicotinamide adenine dinucleotide (NAD)^+^ into NADH [6]. The oxidative reaction of molybdenum is highly dependent on pH and oxygen pressure. In physiological conditions, hypoxanthine and xanthine concentrations in the cell ranged around 1–3 *μ*M, while in hypoxic conditions, the concentrations of hypoxanthine and xanthine increase to about 50–100 *μ*M and cause pH reduction to 7 [6]. In that condition, this enzyme undergoes a posttranslation modification in the 535^th^ and 992^nd^ cysteine residues or proteolysis, resulting in XDH conversion into XO [8].

The electron-transfer affinity (electron flux) of NAD^+^ in the FAD site decreases and the affinity to the oxygen increases during the hypoxic condition. This will cause univalent and divalent electron-transfer generating superoxide or hydrogen peroxide [6, 9]. Therefore, XO is considered one of the peroxide sources in the cells and associated with hypoxic injury [10]. The superoxide generated by the xanthine oxidase could react with nitric oxide (NO) that subsequently will produce peroxynitrite, causing a decrease in NO bioavailability, which then results in reduction in endothelial dysfunction [6]. This alteration is also triggered by the interaction between the positively charged XO and the negatively charged glycosaminoglycan (GAG) on the endothelial surface [11, 12]. Accumulation of XO may also lead to the production of reactive oxygen species (ROS) as superoxide radicals, which could increase the production of hydrogen peroxide triggering endothelial dysfunction [13]. Various reports stated that XO activities can lead to harmful systemic conditions, such as heart attack, chronic obstructive pulmonary disease, pulmonary hypertension, and diabetes mellitus (DM) type 1 and type 2, which are also associated with endothelial dysfunction [6, 14, 15].

The respiratory virus is known to induce the ROS-producing enzymes, including nicotinamide adenine dinucleotide phosphate oxidase (Nox) and XO, as it was observed in influenza virus (IV), human respiratory syncytial virus (HRSV), and human rhinovirus (HRV)-infected respiratory epithelial cells [16]. For example, in bronchial epithelial cells A549 (respiratory epithelial cells) infected by HRSV, it is seen that the XO catalysis product (uric acid) and XO inhibitor (Allopurinol) conversely affect (antagonize) the inflammation process, in this case, the release of proinflammation cytokine and chemokine as neutrophils chemoattractant [2]. The role of XO, the catalysis products (uric acid and superoxide radicals), and XO inhibitors in affecting the inflammation process in respiratory virus infection will be described below.

## 2. Inflammatory Mechanism of Uric Acid due to RNA Virus Infection

Uric acid is expected to trigger IL-1 release through the complex activation pathway of inflammasome and caspase-1 via P2X7 receptor expressed in the surface of the respiratory tract cells, including its lymphocyte [17] and macrophage cells [18]. In incubated human umbilical vein endothelial cells (HUVECs), uric acid is also found to increase the expression of the NLRP3 inflammasome and IL-1*β*, which by the addition of a high concentration of K^+^ and ROS inhibitor results in the reduction of NLRP3 inflammasome expression [19]. This shows that uric acid activates the NLRP3 inflammasome through ROS intermediary or by regulating the K^+^ efflux [19], which is assumed through the interaction of uric acid and P2X7 receptors as its ligands [17, 18]. This, like a study by Neumann et al., shows that the interaction between P2X7 receptors and ATPs besides triggering the inflammasome activity, also causing K^+^ efflux [20]. Moreover, uric acid can also trigger inflammation by activating nuclear factor kappa B (NF-*κ*B) through the signaling pathway of HMGB1/RAGE [21] and by increasing the expression of toll-like receptors (TLR)-4, NLRP3, and ICAM-1 [22]. Inflammatory mechanisms of uric acid are summarized in Table 1.

A study by da Costa et al. showed that NLRP3 inflammasome activation during RNA virus infection is also caused by the host cell structural damage (cytopathogenic) via the intermediary/regulation of K^+^ efflux [24]. A respiratory infection caused by RSV, a negative-single stranded RNA virus, also strongly induces the expression of HMGB1, both *in vitro* and *in vivo* [25]. On the other hand, Pacheco et al. strongly suspected that there is an involvement of P2X7 receptors in the pathogenesis of severe acute respiratory syndrome corona virus-2 (SARS-CoV-2) inflammation [26]. A supportive clinical observation, reported by Huang et al., demonstrated an increase in the number of blood neutrophils in COVID-19 patients when compared to healthy subjects and in intensive care unit (ICU) patients when compared to non-ICU patients [27]. Neutrophils increase the expression of P2X7 receptors and can release IL-1*β* through the activation of NLRP3 inflammasome intermediated by ATP [26] or triggered by the interaction between P2X7 receptors and ATPs [20]. Extracellular ATP is known to be the key mediator in inflammation in lung fibrosis [28]. The existing clinical data, radiography, and autopsies show that patients who suffer from coronavirus infection are at risk for lung fibrosis, and evidence shows that lung fibrosis also occurs in COVID-19 [29]. Based on the above explanation, it can be concluded that there is an inflammatory mechanism between RNA virus infection and uric acid.

## 3. Inflammatory Mechanism of XO and Its Catalytic Products due to Respiratory Virus Infection

Inflammation of host cells, both directly by the virus (through the cytopathogenic activity) and indirectly by the XO activity products, which is uric acid, contributes to the activity of the IL-1*β* cytokine. Research by Fonseca et al. also proved this, where the addition of XO inhibitor (Allopurinol) to the bone marrow-derived macrophages (BMDMs) infected by HRSV showed a decrease in expression and IL-1*β* level compared to without any addition of XO inhibitor [2]. There was an increase in IL-1*β* expression compared to without the addition of uric acid. Uric acid and XO activity were suspected of having important roles during HRSV infection, especially in the initial phase of HRSV infection. It is indicated in the increase of uric acid level in bronchoalveolar lavage fluid of infant mouse infected by HRSV two days postinfection. This increase in uric acid in BALF was also accompanied by the increase in the expression of XO in lung cells.

**Figure 1:**
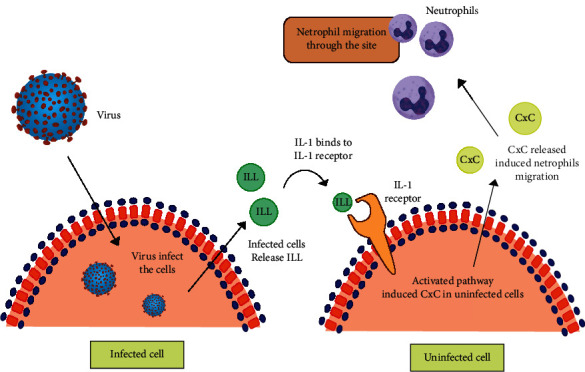
Neutrophils recruitment during RCoV infection. RCoV infects AT1 cells, induces secretion of IL-1, induces CXC in uninfected cells, and triggers neutrophils recruitment (adaptation from Miura et al. [30]).

Respiratory cells inflammation due to respiratory virus infection could more massively occur. Alveoli epithelium is mostly (90%) AT1 cells. The infection of rat coronavirus (RCoV) to the AT1 cells will induce more expression of IL-1*α* and IL-1*β*, which will further send more signals through the IL-1 receptor (IL-1R) of the cells not infected in order to induce more CXC chemokine release. The many numbers of CXC will then trigger more neutrophils to come to the site of the infected cells [30]. The illustration of neutrophils recruitments in RCoV infection is shown in Figure 1.

Neutrophils (and monocyte) recruitment, passing from the endothelium to the infected cells, is significantly contributed to the increase in production of superoxide anion by the Nox2 during infection by the virus [16]. The superoxide anion radicals generated by the activity of the neutrophils during the process of phagocytosis, in the presence of superoxide dismutase (SOD) enzyme, will be converted into hydrogen peroxide (H_2_O_2_) compound. Yasui et al. demonstrated that exogenous SOD can induce neutrophils apoptosis, which means H_2_O_2_ is a mediator inhibiting the inflammation caused by the neutrophils [23]. Although the formation of H_2_O_2_ can inhibit the inflammation through the neutrophil's apoptosis, the next reaction of the H_2_O_2_ also induces an inflammation reaction. By way of Fenton reaction, H_2_O_2_ will react with Fe^3+^ to produce *·*OH radicals. The *·*OH radical is known to have a role in inducing IL-6 cytokine [31], which is a proinflammation agent that appears as a cytokine storm syndrome (CSS) marker, as found in patients with acute respiratory distress syndrome (ARDS) [32].

IL-6 production is also intermediated by uric acid. Cai et al. showed that, similar to the epithelial cells, in endothelial cells, uric acid stimulates the signaling pathway of HMGB1/RAGE, which activates NF-*κ*B. The activation of NF-*κ*B then induces the production and release of IL-6 and TNF-*α* and triggers endothelial dysfunction [21].

Aside from Nox, superoxide anion production is also known to occur due to XO activity in the epithelial and endothelial cells. Fonseca et al. showed that the provision of XO inhibitor (Allopurinol) to HRSV-infected A549 cells indicates a reduced expression of XO, IL-33, and CCL2, compared to the cells without any allopurinol provision [2]. The opposite occurs when HRSV-infected A549 cells are added with uric acid, where there is an increased expression of XO, IL-33, and CCL2, compared to those not added with uric acid. Currently, IL-33 is described as a member of the IL-1 family expressed in large amounts in the epithelial and endothelial cells [33]. It is also described previously that during the infection of RCoV, the interaction between IL-1 and its receptor in epithelial cells of alveolus will induce CXC chemokine secretion [34]. Both CCL2 [34] and CXC [30] are chemokines with roles as neutrophils chemoattractants. A similar condition was also found in rhinovirus- (RV)-infected A549 cells; oxypurinol acts to reduce the production of superoxide radicals, IL-8 and CXCL1 [35]. It can be concluded that XO inhibitors act to reduce neutrophils' recruitment to the infected cells, which eventually can also inhibit the secretion of proinflammation cytokine.

**Figure 2:**
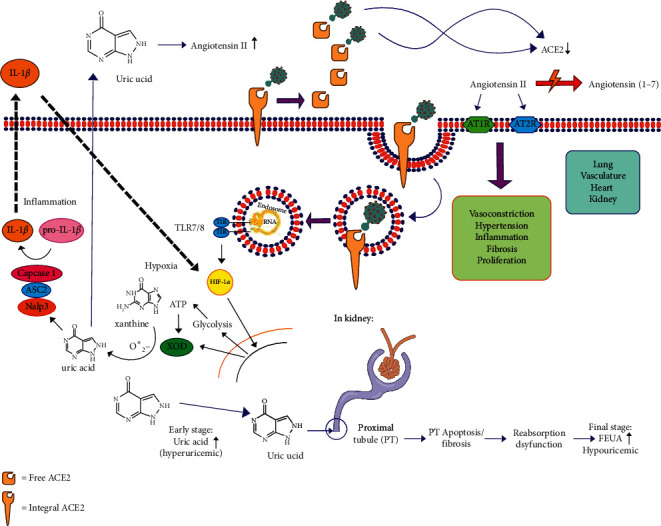
Association between XO activity and uric acid level and kidney tubulus proximal.

According to Chen et al., serum uric acid level >423 mol/L was associated with an increased risk of composite outcome and mechanical ventilation, whereas a level of 278 mol/L was associated with an increased risk of the composite outcome, ICU care, and mechanical ventilation [36]. The condition of hypouricemia can be associated with proximal tubular damage due to viral infection and gout [37]. Damage to the proximal tubule is known from an increase in urinary uric acid levels as happened in SARS patients and COVID-19, which indicates impaired uric acid reabsorption [38]. Thus, it can be hypothesized that during SARS-COV-2 infection, there is an increase in XO activity. It is just that when the amount of uric acid and/or the amount of virus in the circulation increases, hypouricemia can occur as described above. Such a process is described in Figure 2.

## 4. Systemic Impact of Inflammatory Properties of XO due to Respiratory Virus Infection

In the review above, it has been described that during respiratory virus infection, XO and its reaction products, i.e., superoxide anions and uric acid, have important roles in the formation of proinflammation cytokine in macrophage cells [2], epithelial cells [2, 30], and endothelial cells [19, 21]. Proinflammatory cytokine formed during the respiratory virus infection are IL-1, TNF-*α*, and IL-6. As is the case with COVID-19, there is an increase in IL-1*β*, IL-6, and C-reactive protein (CRP) [39]. Susilo et al. reported that proinflammation cytokine, i.e., TNF-*α*, IL-1, and IL-6, also IL-8, infection markers such as procalcitonin, ferritin, and CRP, are also found higher in severe COVID-19 [40].

During respiratory virus infection, XO also acts to induce chemokine secretion (CXC, CXCL1, and CCL2, among others) that are neutrophils chemoattractants [2]. In COVID-19 patients with ARDS, hyperinflammation occurred due to the proinflammation cytokine release in high numbers and also due to the secretion of chemokine in large numbers (CCL2, CCL3, CCL5, CXCL8, CXCL9, and CXCL10, among others) [40]. The release of chemokine in large numbers will enhance the recruitment of neutrophils, increase the number of superoxide anions produced during the phagocytosis process by the neutrophils, which will eventually raise the number of proinflammation cytokines secreted and worsen the inflammation reaction.

Neutrophils' recruitment to the inflammatory sites may become key that explained severity in viral infections, such as influenza, and not limited to SARS-Cov2 [41]. Neutrophils migrations through the closest endothelial near infected cells such as alveolar epithelial cells may cause induced neutrophils apoptotic, neutrophils extravascular trap (NET), and accumulation together with platelets in the endothelial cells [42]. Such a phenomenon may lead to microthrombus formation and may trigger lung hypoxic damage [43–45]. It was mostly a similar mechanism to pneumonia cases in COVID-19 [46].

It is explained in the previous paragraph that during the respiratory virus infection, hyperinflammation especially occurs due to neutrophils recruitment in uninfected cells. The same thing also happens during a gout attack. Neutrophils' recruitment when responding to the presence of monosodium urate crystals is the key factor in acute inflammation or hyperinflammation [47]. However, it is often found that gout attack and inflammation reactions may happen in patients with normal serum uric acid levels. A retrospective study to 30 patients who had gout attacks conducted by Badulescu et al. found that 63.3% of patients had normal serum uric acid levels [48]. Zhao et al. even said that during a gout attack, 34.92% of 126 patients had a reduction in serum uric acid level} [44]. Badulescu et al. also said that the inflammation syndrome was detected in 76.6% of patients with a high erythrocyte sedimentation rate (ESR) [49]. It can be concluded that patients with normal serum uric acid levels can also have gout attacks and an increase in ESR, which are the marker of the occurrence of inflammation. Soluble uric acid is also known to be able to activate NLRP3 inflammasome [50]. A normal uric acid level or less than 6 mg/dL can also trigger hyperinflammation. In respiratory virus infection, excessive recruitment of neutrophils seems also influenced by the many cells infected.

The increase in ESR is also commonly found in COVID-19 patients, in up to 85% of total hospitalized patients [51]. It is very likely that this increase in ESR is not followed by the increase in serum uric acid level, as happens in patients having an acute gout attack. Neutrophils to lymphocyte ratio monitoring can also be done and can be used as an indicator of hyperinflammation. Serum uric acid level monitoring may be conducted particularly on COVID-19 patients with ARDS. Lee et al. [52] and Elshafey et al. [38] mentioned that in patients with ARDS, the serum uric acid level is a mortality risk prognosis marker. Serum uric acid in low levels in ARDS patients will usually result in clinical improvements. In contrast, serum uric acid level above the threshold value of 8.4 mg/dL is known to be associated with the mortality of ARDS patients [38].

Excessive superoxide radical formation can also damage lymphocyte T cells [53] via necrosis [54] that can trigger lymphopenia. Lymphopenia is known to occur in more than 80% of COVID-19 patients [40], marked by the decrease in circulating CD_4_^+^ T cells and CD_8_^+^ T cells [55, 56]. The low level of CD_4_^+^ cells leads to the decrease in neutralizing antibodies and gives impact to the disturbance in the immune system balance. The decrease in the cytotoxic CD_8_^+^, however, causes weak antibody production and inefficient virus clearance [54, 55]. Lymphopenic T cells also have an impact on the reduction of apoptosis ability of phagocytes, which also causes excessive immune response [39].

Excessive immune response, or CSS, caused by cytokine proinflammation, chemokine, and neutrophils production can trigger an acute condition in COVID-19 or ARDS patients. An excessive immune response can also lead to multiorgan failure (MOF), such as lung damage and fibrosis due to endothelial dysfunction. It is already shown that inflammation reaction by XO or uric acid activity can trigger endothelial dysfunction [13, 21].

Endothelial dysfunction by XO is initiated when XO in the circulation bind to the sulfated GAG in the surface of endothelial cells [7, 11, 57], catalyzing the formation of uric acid and superoxide radicals, triggering the production of proinflammation cytokine [19, 21], which then independently trigger cell apoptosis [38], disturbing the permeability and the function of cell barrier [58], and finally causing the endothelial dysfunction. Consistent with the above, it is known that XO inhibitor will improve endothelial function, such as in patients with coronary artery disease, chronic heart failure, and DM1 and DM2 [39]. In COVID-19 patients, hypertension and diabetes are comorbid diseases that increase the risk of SARS-CoV-2 infection [40]. In accordance with World Health Organization data, cardiovascular diseases, diabetes, hypertension, chronic respiratory disease, and cancer are comorbid diseases found in fatal cases in COVID-19 patients [39]. In view of the above, the inhibition of XO activity, including in cases caused by a respiratory virus such as SARS-CoV-2, can be used as the target of therapy, especially to maintain the clinical conditions of COVID-19 patients so that the comorbid diseases do not develop into a serious complication that can lead to MOF and resulting in death.

## 5. Xanthine Oxidase (XO) as a Therapeutical Target in Hypoxia in COVID-19

One study showed that SARS-CoV-2 infections induced formation NET [59]. These extracellular fibers consisted of neutrophils's DNA that binds to pathogenic agents such as viruses and bacteria [60]. However, NETs also beheld undesirable effects to the host, such as induced inflammations, endothelial damage, and microthrombus formation [61]. NETs were also reported to contribute to coagulopathy during HIV infections and play a role during stroke related thrombocyte [62, 63]. Moreover, a meta-analysis study that assessed histological findings of COVID-19 patients showed that lung damage in patients were caused by alveolar blood-air barrier damage, in parallel with hyaline membrane formation and microthrombus in vein of alveolar vascularate [64]. Therefore, we assumed such incidence that SARS-CoV-2 infections induced NET formations.

**Figure 3:**
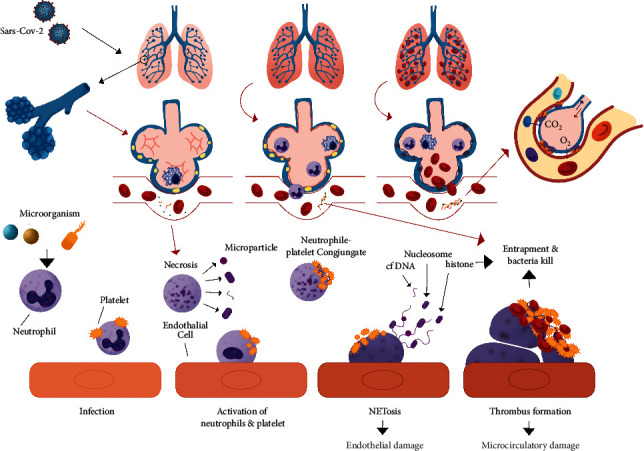
mechanism that explained SARS-CoV-2 infections NETosis and relations to hypoxic cell damage. The mechanism was adapted from Zhou et al. and Khafarijiy et al. [65, 66].

In COVID-19 patients, ARDS may generally be triggered by alveolar vasoconstriction. Vasoconstriction process may occur due to microthrombus formation in pulmonary capillaries as a response to alveolar hypoxia and an increase in the amount of angiotensin II. As a result, there is inhibition of ACE2 activity by the virus. This causes pulmonary blood flow to flow persistently with high pressure to the alveoli afterward. Furthermore, this may cause an increase in blood vessel pressure in the lung, which later causes vascular fluid leakage into the alveoli and causes edema. Increased edema will cause atelectasis and will trigger pulmonary right-to-left shunting, resulting in an incomplete gas exchange in the alveolus. As a result, oxygen saturation will continue to decrease, and hypoxemia persists even though the administration of the oxygen fraction is increased through oxygen supplementation [45]. This is shown in Figure 3.

Even though ARDS may be induced by alveolar vasoconstriction appears in some patients, recent evidence shows dilatation on pulmonary capillaries [67]. This may be associated with inflammation and endothelial damage due to NET, as described above. Loss of vasoconstriction regulation in response to hypoxia seems to explain the findings of COVID-19 patients without symptoms of shortness of breath (dyspnea). Apart from inflammation in endothelial cells, pulmonary vasoconstriction dysregulation can be associated with an increased amount of VGEF-A, a protein that can mediate vascular dilation, in bronchial alveolar lavage fluid from COVID-19 patients [68]. It is known that viral spike proteins can bind to neuropilin-1, which is a VEGF-A coreceptor and is expressed on pulmonary vascular endothelial cells [69]. This causes VEGF-A to tend to bind to VEGFR-1 and VEGFR-2, which are also expressed on pulmonary vascular endothelial cells and mediate vasodilation.

Even though ARDS triggered by pulmonary hypertension appeared in some patients, a common incidence that may show alveolar vasodilatation [67], which is related to endothelial damage and inflammation due to NETosis as described before. Loss of vasoconstrictions regulations as hypoxia response may be described in asymptomatic COVID-19 patients without dyspnea, commonly dubbed as “happy hypoxia.”

**Figure 4:**
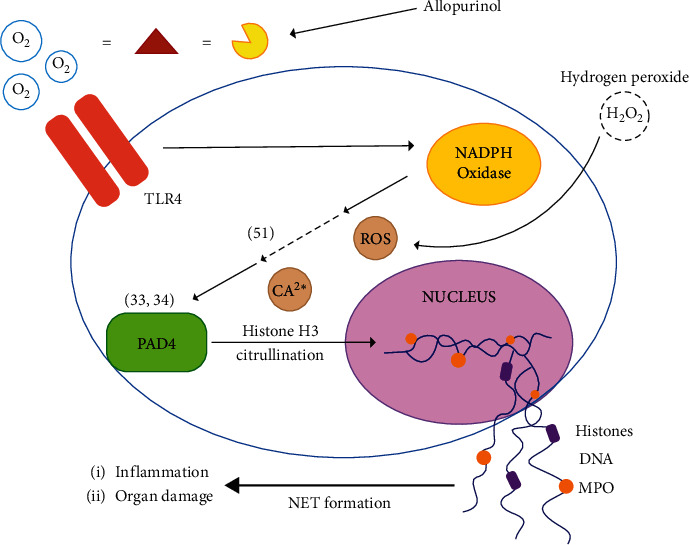
Allopurinol suppressed NETosis formations in vitro. XO activity triggered NETosis in vitro while administration of allopurinol inhibits NETosis. The figure was adapted from Khafarijiy et al. [66].

In summary, SARS-CoV-2 may trigger NET that causes hypoxic cell damage due to contribution of that radical superoxides that produced by XO. Al-khafaji demonstrated that neutrophils incubated with XO and hypoxanthine, triggered NET formation. Sequentially, allopurinol administrations to neutrophils show NET formations. Therefore, this may show that allopurinol may be able to reduced NETosis, which may be caused by reduced expression of citrullinated histone H3 (Cit-H3) [66]. Such processes were shown in Figure 4.

Viral infection has been shown to induced XO activity in RSV infections. (2) Uric acid, which is produced by RNA, may play a role in IL-1b formations as shown by Nicholas et al. [70]. Moreover, hypoxia-inducible factor 1 (HIF-1) as hypoxia biomarkers may also contribute to such mechanism [70]. This may also be related to the role of ATP in sequential reactions of IL-1b formation and release, which is a sign of increased catabolic activity by the cells. As previously described in Figure 1, CXC, which attracts neutrophils [30], may induce NET formations, hyperinflammations, damage of alveolar capillary endothelial, and microthrombus formations, which later caused hypoxemia as described previously [59, 61, 66]. Therefore, XO and its catalytic product, uric acid, and superoxide radicals may trigger hypoxic cell damage in COVID-19.

In several studies, uric acid may become a prognosis marker of ARDS, which is caused by the buildup of alveolar fluid [71]. In contrast, in several cases, COVID-19 has conditions of normourisemia or hypouricemia. Werion et al. explained that such conditions may be caused by kidney tubules proximal that have been damaged, which may disrupt uric acid absorption. This may be indicated by an increased level of urine uric acid and fractional excretion uric acid (FEUA) in COVID-19 patients [72]. Koitka et al. also stated that ACE2 were expressed sufficiently in kidney proximal tubulus [73], therefore may be susceptible to SARS-COV-2 infections. Moreover, uric acid has been demonstrated to induce apoptosis in kidney tubules proximal [74]. Both facts may explain hipo/normosermia in COVID-19 patients.

A better perspective of the uric acid drug may be used in COVID-19 therapy as reported by Tardif et al. [75]; at this report, colchicine as a uric acid drug has been reported to reduce 25% of patients to be hospitalized, 50% of patients that need a ventilator, and 44% of deaths [75]. Colchicine has been known to inhibit neutrophils to inflammations sites caused by uric acid crystals. Therefore, allopurinol, which has the same mechanism as colchicine, may potentially behold as having a similar effect to reduced neutrophils recruitment and prevent NETs related to endothelial cells.

Lastly, colchicine and allopurinol are cheap drugs that are more accessible in markets [76], with better-known side effects. Therefore, behold other potential applications to these drugs. However, correct administrations protocols as second lines to that COVID-19 patients should be studied. For allopurinol, a clinical trial should be performed.

## 6. Conclusions and Future Prospect

Control in the formation of proinflammation cytokines causing inflammation is a key factor in disease therapy caused by a respiratory virus infection, including also the disease caused by SARS-CoV-2. In this case, the use of a combination of anti-inflammation and antivirus may be more effective than only using one single modality. The failure in controlling the inflammation reaction during respiratory virus infection can lead to endothelial dysfunction resulting in MOF and CSS leading to ARDS. It is also already shown that XO activity has an important role in inflammation reaction during respiratory virus infection. Therefore, XO can be used as the target of anti-inflammation therapy caused by respiratory viruses, including also COVID-19.

XO and its catalytic product are potential therapeutic targets in COVID-19 patients, in particular during the early phase of infections or while symptoms appear. Uric acid may also be potential for monitoring inflammation and hypoxia in COVID-19 patients during the early step of infections in patients with uric acid comorbidity or others influenced by uric acid conditions.

## Figures and Tables

**Table 1:** Inflammatory mechanism of uric acid.

Secretion/expression	Cell	Mechanism	Reference
IL-1	Lymphocyte	Activation of inflammasome and caspase-1 complex via P2X7 receptor	[17]
IL-1*β*	Macrophage	Activate the inflammasome via P2X7 signaling, and treatment with a P2X7 inhibitor reduces IL-1*β* release	[18]
IL-1*β*	Human umbilical vein endothelial cells (HUVECs)	Regulate the activation of NLRP3 inflammasome by activation of ROS and K + efflux	[19]
IL-6 and TNF-*α*	Human umbilical vein endothelial cells (HUVECs)	High concentrations of UA significantly increased mRNA expression and extracellular release of HMGB1 from human umbilical vein endothelial cells (HUVECs) [23]. Extracellular HMGB1 binding to RAGE activates NF-*κ*B, which leads to proinflammation.	[21]
IL-1*β*	Renal tubular epithelial cells (HK-2)	UA, like Lipopolysaccharides (LPS), significantly enhanced the expression of TLR4, NLRP3, IL-1*β,* and ICAM-1	[22]
